# Hair cell alteration prevalence rates in students of a school in Distrito Federal

**DOI:** 10.1590/S1808-86942012000400017

**Published:** 2015-10-20

**Authors:** Valéria Gomes da Silva, André Luiz Lopes Sampaio, Carlos Augusto Costa Pires de Oliveira, Pedro Luiz Tauil, Glaucia Magalhães Brito Jansen

**Affiliations:** 1 MSc (Speech and Hearing Therapist); 2 PhD (Associate MD in the Cochlear Implant Department of the University of Brasília, Brazil); 3 PhD (Professor of the University of Brasilia, Brazil); 4 PhD (Professor in the Graduate Program on Tropical Medicine of the Medical School of the University of Brasilia); 5 Graduate student, Audiology (Speech and Hearing Therapist)

**Keywords:** adolescent health, hair cells, auditory, outer, hearing loss, noise-induced

## Abstract

Exposure to loud music is increasing among young people, and so could be the number of hearing impairment cases in this population. Otoacoustic emission tests are sensitive in capturing the effects of exposure to noise, and allow the detection of early cochlear disorders.

**Objective**: This study aims to look into the prevalence rates of injuries to outer hair cells in a population of students through otoacoustic emission testing.

**Materials and Method**: One-hundred and thirty-four subjects were submitted to transient evoked and distortion product otoacoustic emission tests. Subjects were assessed on a “pass/fail” scale. This is a cross-sectional descriptive study on prevalence rates.

**Results**: More than four fifths (80.6%) of the 134 subjects had altered transient otoacoustic emissions, most of whom were males; 97.8% had altered distortion product otoacoustic emissions and 79.9% had altered test results in both transient evoked and distortion product OAEs; most were males; 94.0% reported they used earphones; and 82.8% stated they frequented places where loud music was played.

**Conclusion**: The high prevalence rates of altered test results seem to indicate the presence of early cochlear disorders in the studied subjects. A significant number of subjects reported exposure to loud music, a habit that may be conducive to the onset of cochlear disorders.

## INTRODUCTION

A lot has been said in the media about hearing and possible cases of hearing loss in young people connected to exposure to noise.

Young people are increasingly exposed to noise. What many may not know is that even sporadic exposure to noise can produce significant harm and alter a person's physical and mental welfare^1^.

The human hearing apparatus is extremely vulnerable to noise. In high intensity levels, noise may impair hearing and one's general health status^2^.

Among the agents noxious to hearing, noise is considered a significant factor in the increasing rates of hearing impairment and one of the main causes for sensorineural hearing loss in adults^3^, ^4^, ^5^. Young populations are still being studied.

The idea that hearing loss by exposure to noise is connected only to adults (the elderly) or that it is specific to occupational circumstances must be reviewed. The audiograms of young people with hearing loss share the same characteristics of audiograms of people with noise-induced hearing loss^6^, ^7^.

The use of earphones is widespread, especially among young people who use them for entertainment and leisure. Other practices appreciated by young people such as listening to loud music, wearing cell phone or MP3 player earbuds, going often to music concerts, clubs, and gyms may be harming their auditory health. These activities may be pleasurable, but are deemed noxious to hearing when performed without moderation^8^.

Assessment and monitoring of hearing loss is usually done through pure-tone audiometry. However, otoacoustic emissions (OAEs) are more sensitive for adverse events related to exposure to noise and allow the early detection of cochlear alteration before pure-tone audiometry. OAEs enable the specific assessment of outer hair cell function. OAE tests are non-invasive, sensitive for cochlear status, and do not introduce discomfort or risks to the patient: they are easy to perform, painless, widely applicable^2^, ^9^, ^10^, ^11^.

Considering the effects of noise upon the cochlea and the preventive character of evoked otoacoustic emissions in auditory monitoring, this study was carried out to assess outer hair cell function through the examination of otoacoustic emissions in a group of students from Distrito Federal to find the prevalence rates of altered test results in the sample.

## MATERIALS AND METHODS

This cross-sectional descriptive study on prevalence rates was approved by the Ethics Committee and given permit n^o^ 060/2008.

A sample of 144 middle school students of both genders from a private school in Distrito Federal was randomly selected between April and May of 2010. Ten individuals were excluded for middle ear alterations. Therefore, the data collected reflect the reality of 134 subjects, 56 males and 78 females with ages ranging between 14 and 19 years.

The enrollment criteria included: absence of otological complaints and symptoms; no history of hearing loss; not taking ototoxic medication; not using hearing aids; not having middle or outer ear problems such as ear wax and infection. A questionnaire was given to study prospects to find out where subjects stood on matters pertaining to enrollment criteria, their hearing habits, ear diseases, hearing loss and others.

Data collection took place at the student's school. Their ear canals were inspected for ear wax and other findings that could interfere with test procedures. Subjects were then submitted to transient evoked and distortion product OAE tests on a portable MAICO device model ERO-SCAN made in 2006. Tests were done in a quiet environment and right ears were tested first.

Transient evoked OAE testing considered normal or “Pass” results when amplitudes were equal to or greater than -12 and signal to noise ratios were equal to or greater than 6 dB on all six tested frequencies (1.5 kHz-4 kHz). Distortion product OAE testing considered normal or “Pass” results when amplitudes were equal to or greater than -5 and signal to noise ratios were equal to or greater than 6 dB on all six tested frequencies (2 kHz-12 kHz). The results of both TEOAE and DPOAE were analyzed together and results were categorized as “FAIL” when alteration on at least one ear was seen on either of the tests.

The variables studied for statistical analysis purposes were signal amplitude, signal to noise ratio, gender, and ear side. Results were reported in the form of mean, minimum and maximum values, standard deviation, and absolute values (n).

The possible differences in the mean ages of subjects of each gender were investigated using Student's *t*-test. The chi-square test (Fisher's exact test) was used to analyze the prevalence rates seen in TEOAE and DPOAE tests for criterion “pass/fail” for gender and both ears.

The comparisons between mean amplitudes and signal to noise ratios for genders, each ear and frequency were performed using the mixed design ANOVA test with factors gender (two levels, factor between subjects), ear (two levels, repeated measurement) and frequency (six levels). The mean values of the results were checked for the existence of statistically significant differences. The Greenhouse-Geisser method was used to correct sphericity deviations. Post hoc analysis was done to look into interactions between up to two factors.

The correlation between prevalence of failed results for signal amplitude on each ear and frequency analyzed on TEOAEs was assessed through Pearson's correlation. The same test was used to analyze the signal to noise ratio on DPOAEs.

The association between prevalence of failed results on both tests (TEOAE and DPOAE) and gender was analyzed using the chi-square test (Fisher's exact test). The same test was used to assess the association between gender and exposure to loud music. Statistical significance level was set at 5% (*p* = 0.05). All tests were bicaudal.

In order to allow the study of prevalence rates, the size of the sample was calculated for a 95% confidence interval, and the estimated prevalence for OHC alteration of 90% resulted in a sample of 121 subjects.

## RESULTS

The analysis of TEOAE results revealed that only 19.4% (26) participants passed the test with both ears; 29.9% (40) passed only with their left ears; 29.1% (39) passed with their right ears; 80.6% (108) failed on both ears; 70.1% (94) failed on their left ears; and 70.9% (95) failed on their right ears.

Considering gender, TEOAE results showed that 28.2% (22) of the females enrolled in the study passed the test and 71.8% (56) failed. In the male group, 7.1% (4) passed and 92.9% (52) failed. In the entire group of subjects, 80.6% (108) failed the TEOAE test.

Statistically significant difference was found for variable gender and presence of altered TEOAE test results. The percentage of males failing the test was significantly greater than the percentage of females (*p* = 0.003).

**Figure 1:**
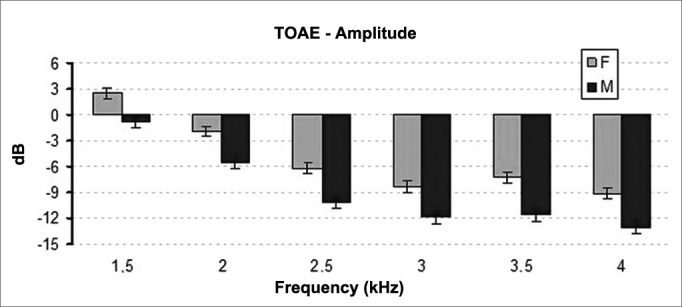
Mean and standard deviation values of the TOAE amplitudes recorded for each gender in each frequency. F: Female; M: Male. ANOVA Test, *p* < 0.001.

Statistically significant difference was found when the distribution of signal amplitude on TEOAE was analyzed for gender. Female participants had greater levels of signal amplitude than their male counterparts (*p* < 0.001) (Figure 1).

**Figure 2:**
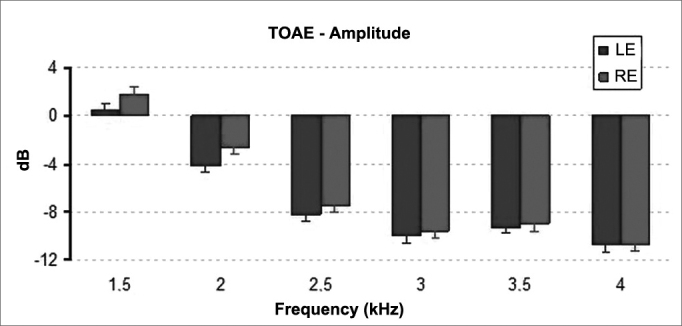
Mean and standard deviation values of the TOAE amplitudes recorded for each ear in each frequency. LE: Left Ear; RE: Right Ear. ANOVA test, *p* = 0.009.

ANOVA for repeated measurements revealed statistically significant differences between mean signal amplitudes of right and left ear TEOAE results (*p* = 0.009). On average, the amplitudes recorded for right ears were greater than the values for left ears (Figure 2).

**Figure 3:**
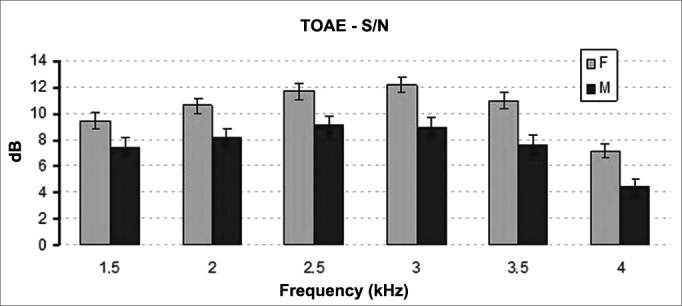
Mean and standard deviation values of the signal/noise ratio of the TOAE recorded for each gender in each frequency. S/N: signal/noise ratio; F: Female; M: Male. ANOVA test, *p* < 0.001.

**Figure 4:**
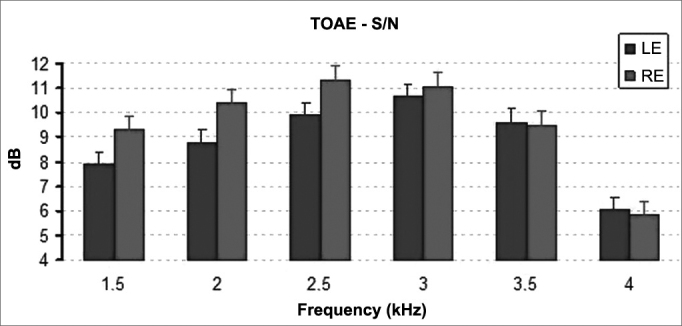
Mean and standard deviation values of the signal/noise ratio of the TOAE recorded for each ear in each frequency. S/N: signal/noise ratio; LE: Left Ear; RE: Right Ear. ANOVA test, *p* = 0.022.

The distribution of signal to noise ratios on TEOAE tests elicited statistically significant differences between genders. Female participants had higher signal to noise ratios than males (*p* < 0.001) (Figure 3). Statistically significant difference was also seen between the signal to noise ratios recorded for right and left ears **(*p*** = 0.022). Right ears had significantly higher mean signal to noise ratios (Figure 4).

The analysis of percent failed TEOAE test results for signal amplitude on each frequency and for each ear revealed a positive linear correlation between frequency and percent failed test results on left ears (*p* = 0.004) and right ears (*p* = 0.003), stressing a trend in which the higher the frequency the higher the percentage of failed tests. Likewise, the assessment of signal to noise ratios also revealed a relationship between frequency and percent failed tests on both ears. On left (*p* = 0.042) and right ears (*p* = 0.001) a trend of increased number of failed tests was observed as frequencies were increased.

The “pass/fail” analysis of distortion product otoacoustic emission tests showed that 2.2% (3) participants passed on both ears; 8.2% (11) passed on left ears; and 7.5% (10) passed on right ears. However, 97.8% (131) failed on both ears; 91.8% (123) failed on left ears; and 92.5% (124) on right ears.

The analysis of gender and DPOAE test results revealed that none of the male participants passed and only 3.8% (3) females passed. Among the failed DPOAE tests, 97.8% (131) of the participants failed in at least one ear. There was no statistically significant difference between genders and presence of altered DPOAE test results (*p* = 0.256).

The analysis of signal amplitudes for each frequency showed that the lower values for left ears were found in the 8 kHz and 12 kHz bands. The signal to noise ratios for left ears were within normal standards, except for the 12 kHz band. Right ears showed similar results when mean amplitudes and signal to noise ratios for each frequency were analyzed. Lower amplitude values were also observed in the 8 kHz and 12 kHz bands and in the 12 kHz for signal to noise ratios. On right ears, amplitude and signal to noise ratios at 12 kHz were altered.

**Figure 5:**
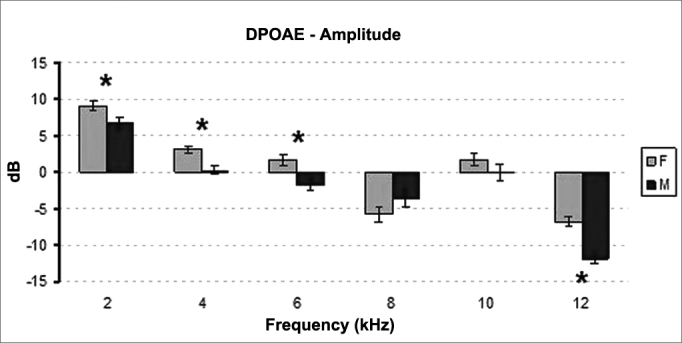
Mean and standard deviation values of the DPOAE amplitudes recorded for each gender in each frequency. F: Female; M: Male. ANOVA Test, *p* < 0.007.

Statistically significant difference was found when the distribution of signal amplitudes on DPOAE between genders was compared. Females had higher signal amplitude than males (*p* < 0.007) (Figure 5).

**Figure 6:**
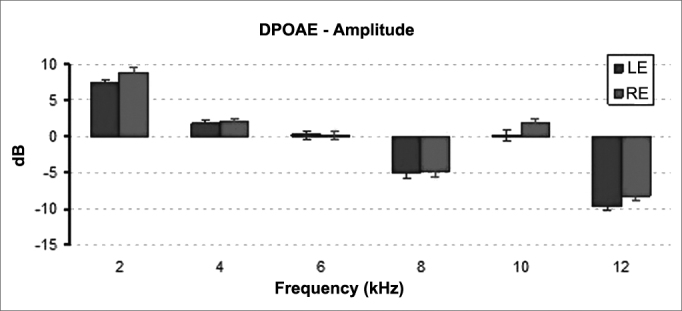
Mean and standard deviation values of the DPOAE amplitudes recorded for each ear in each frequency. LE: Left Ear; RE: Right Ear. ANOVA test, *p* = 0.017.

Statistically significant difference was also found in the DPOAE mean signal amplitudes of right and left ears (*p* = 0.017). The mean signal amplitudes recorded on right ears were significantly higher than the values see on left ears (Figure 6).

**Figure 7:**
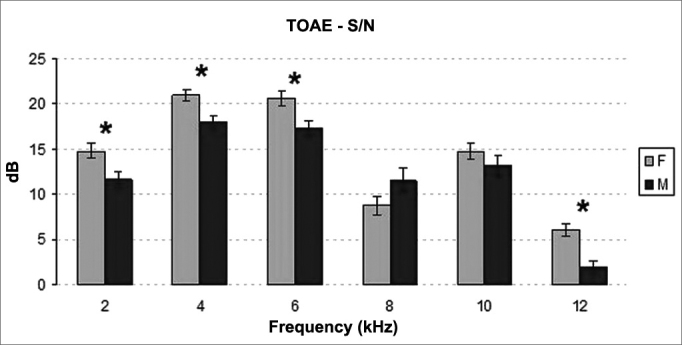
Mean and standard deviation values of the signal/noise ratio of the TOAE recorded for each gender in each frequency. S/N: signal/noise ratio; F: Female; M: Male. ANOVA test, *p* < 0.013.

**Figure 8:**
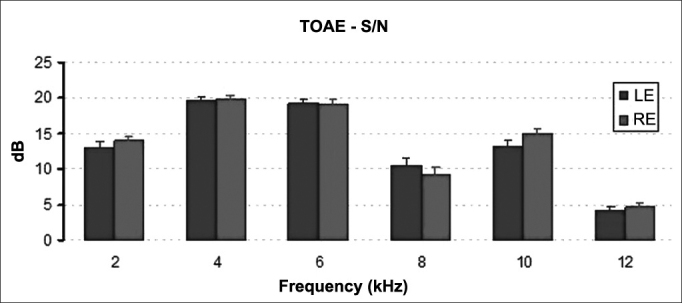
Mean and standard deviation values of the signal/noise ratio of the TOAE recorded for each ear in each frequency. S/N: signal/noise ratio; LE: Left Ear; RE: Right Ear. ANOVA test, *p* = 0.499.

The distribution of signal to noise ratios on DPOAE tests elicited statistically significant differences when genders were compared. Female subjects had higher signal to noise ratios than males (*p* < 0.013) (Figure 7). No statistically significant difference was found between ears (*p* = 0.499) (Figure 8).

The analysis of DPOAE failed tests and signal amplitude on each frequency and ear side showed a statistically significant correlation between frequency and failed tests on left ears (*p* = 0.008) and right ears (*p* = 0.003), revealing that the higher the frequency the greater the number of failed tests. The same trend was observed in the analysis of signal to noise ratios. Both right and left ears had more failed tests in higher frequencies.

The combined analysis of transient evoked and distortion product otoacoustic emissions resulted in the following: 79.9% of the 134 (107) failed both on TEOAE and DPOAE tests in at least one ear. In the female group (78), 29.5% (23) passed and 70.5% (55) failed. In the male group (56), 7.1% (4) passed and 92.9% (52) failed.

The possible association between gender and failed test results was tested. The chi-square test revealed that more males failed the test than females (*p* = 0.002).

In terms of exposure to loud music, 94.0% of the 134 (126) wear earphones and 82.8% (111) reported they frequented places where loud music was played. There was no statistically significant correlation between gender and use of earphones (*p* = 0.278), gender and frequenting places where loud music is played (*p* = 0.163), or use of earphones and frequenting places where loud music is played (*p* = 625).

## DISCUSSION

Some authors have made reference to the sensitivity of TEOAEs in detecting subtle cochlear alterations from exposure to noise^2^, ^12^, ^13^. The presence of TEOAEs suggests that most thresholds is within normal standards and, conversely, their absence may indicate OHC involvement^14^. Our subjects are young and assumedly have normal hearing, thus the sensitivity of TEOAE assessment was a considerable factor.

A lower number of alterations was expected. Given that the subjects were not assessed through audiometry and assuming their hearing thresholds are normal, the percentages found showed that the absence of TEOAEs may occur even in patients with supposedly normal hearing thresholds.

Other studies have found higher percentages of normal results than ours, but the assessment criteria adopted by their authors was less strict as they took only the signal to noise ratio into account^15^.

Amplitude records in the TEOAE test signal of both ears had negative values for the most part. Consequently, the mean amplitude values in both ears were also negative in almost all frequencies, except for the 1,500 kHz band.

The consistently negative values observed in TEOAE test signal amplitudes were inferred to be a characteristic of the energy of the TEOAE spectrum measured by the device used in this study, given the recommended amplitude minimum negative value of -12 dBNPS. Other authors have also observed negative amplitude values in TEOAE tests^15^, ^16^.

TEOAE amplitude may vary as a function of age, gender, and ear side. It may also be affected by the level of external ambient noise or internal (individual) noise, and stimulation sound pressure levels. The higher the age the lower the response amplitude; in neonates amplitude values reach about 20 dB, 10 dB in young adults, and 6 dB in elderly subjects^17^. This aspect was not considered in this study, as subjects were within closer age ranges.

A correlation between increases in frequency and decreases in signal amplitudes was observed in this and in another study^15^. The decrease in TEOAE amplitudes at higher frequencies may be related to middle ear filtering properties and short latencies seen in higher frequencies, which hamper recording through the device's microphone^18^.

No other references with the same analysis criteria or with negative TEOAE amplitudes were found in the literature. Thus, the description of the findings of this study may serve as a source of data for other studies in which the total energy of the signal amplitude spectrum is analyzed or in which the same OAE measurement device is used.

The comparison of the mean amplitude values seen in TEOAE signal tests for right and left ears revealed that higher amplitude values were recorded in right ears and female subjects, a finding consistent with the literature^17^.

Some authors have discussed that higher right ear TEOAE amplitudes may be related to the impact of spontaneous otoacoustic emissions; SOAEs are generally bilateral, but when they manifest unilaterally they are more frequently seen in right ears^17^, ^19^. However, in the references included in this study, asymmetric TEOAE measurements is not mentioned. In general terms, TEOAEs behaved symmetrically in both ears^2^, ^14^, ^20^, ^21^, ^22^.

The 1 kHz band was not included in this study. Frequencies starting from 1.5 kHz were included, and the highest mean amplitudes were observed at 1.5 and 2 kHz. According to the literature, greater TEOAE amplitudes in adult subjects reside between 1 and 2 kHz, while in neonates they occur between 3 and 4 kHz. These differences are attributed to the effects of size of the outer and middle ear, to the resonance characteristics of the external acoustic meatus, and the presence of spontaneous OAEs reinforcing certain frequencies^17^.

In this paper, the values recorded in signal to noise ratios were positive for most frequencies. As seen in TEOAE amplitudes, the signal to noise ratios were different at 4 kHz in both ears. Lower signal to noise ratios were observed at 4 kHz, supporting the idea that it is difficult to record TEOAEs in higher frequencies due to middle ear properties and the shorter latencies seen in these frequencies^18^.

DPOAEs also showed a high number of failed tests. The prevalence rate of altered distortion product otoacoustic emissions in right ears was 92.5% and 91.8% in left ears, leading to a combined total of 97.8% of failed results.

Another study performed on subjects exposed to loud music after sports activity revealed a higher percentage of normal results (75%) than ours (2.2%). However, the assessment was not as precisely described^23^.

A study done on adolescents revealed a significant percentage of altered results (63%)^24^. The rates reported in this study were higher than the rates reported in other studies, and significant differences were also seen in the findings of this study when compared to others cited herein, possibly due to methodological differences.

It has also been considered that this difference may be due to the use of stricter criteria and the assessment of high frequencies in this study, given that more altered results were seen at 12 kHz. However, this finding may indicate earlier cochlear disorder.

TEOAE mean amplitudes were reduced as frequencies were reduced, as also seen in other studies^15^, ^25^. Other authors have reported difficulty recording high frequencies in TEOAEs^18^. It is believed that in the case of DPOAEs there is a correlation between short latency at high frequencies and difficulty capturing DPOAEs through the otoacoustic emission analyzer's microphone.

In this study, the mean amplitudes in right ears were significantly greater than the values seen in left ears as. As seen on TEOAE amplitudes, when left and right ears were analyzed the DPOAE amplitudes at 8 and 12 kHz were significantly lower than the amplitudes seen in other frequencies.

The idea that lower amplitudes could be indicative of early cochlear involvement was considered, once lower signal amplitudes and signal to noise ratios occur at higher frequencies. And it may be considered that such cochlear areas may be affected by noise^26^. Despite the lack of other justifications for this fact, this data cannot be considered by itself. Further studies need to be carried out to look into records of DPOAE absolute amplitudes in subjects using high frequencies (above 8 kHz).

Mean signal to noise ratios were higher in frequencies below 12 kHz, except for the 8 kHz band. In both ears, mean signal to noise ratios were lower at 12 kHz (4.1 on left ears and 4.5 on right ears).

Therefore, this finding may be related to the onset of early cochlear involvement in adolescents, given that another study^27^ also found that higher frequencies were more affected by the deleterious impact of exposure to noise.

As done on TEOAEs, the prevalence of failed results on DPOAEs was analyzed for ear side and gender in connection to the verification of two parameters: signal amplitude and signal to noise ratios in all six frequency bands.

Frequency is a more specific parameter in DPOAEs, and cochlear function can be assessed from the basal to the apical turn of the cochlea. Response at lower frequencies is harder to measure due to the presence of ambient and internal noise, as seen by the lower signal to noise ratio at 2 kHz. These emissions provide for more accurate information at higher frequencies (above 2 kHz). Responses at 8 kHz are generally not good. In this scenario, a loudspeaker with more voltage - and thus more distortion - is needed^17^. This study observed decreased responses at 8 kHz, possibly due to the reasons mentioned above.

The combined TEOAE and DPOAE test results showed a surprisingly high number of “Fail” ratings (79.9%), with little variation between ears. The analysis of the data in this study revealed that the percent occurrence of alterations in both TEOAE and DPOAE tests may vary significantly. It is assumed that this variability may be connected to methodological aspects, to the parameters used, and the criteria selected for analysis.

The supposed alterations on outer hair cells found in the TEOAE and DPOAE tests are not sufficient to change audiometric thresholds; injuries in up to 30% of the outer hair cells with preserved inner hair cells may occur before any hearing loss is detected^19^, ^28^.

Therefore, OAEs are effective in assessing early cochlear function (OHC) in subjects exposed to noise without a diagnosis of hearing loss. It is recommended that this test be added to the clinical routine to aid in the diagnosis of cochlear alteration in subjects exposed to noise, as is the case of individuals exposed to loud music.

In this study, the results related to exposure of adolescents to loud music suggest that the youth seems to be unworried about the harmful effects of loud music as confirmed by the high prevalence of exposure to loud music.

Almost all adolescents reported they had the habit of wearing earphones and frequenting places where loud music was played. One hundred and twenty-six (94.0%) individuals said they wore earphones and 111 (82.8%) reported going to places where loud music was played. Another study described lower rates of individuals wearing earphones (76%) when compared to subjects going to places where loud music was played (91%)^24^.

Despite the pleasures connected to listening to music, it may become a source of harm. Agreeable sound such as music is less harmful than sounds considered unpleasant such as industrial/occupational noises, but may still be a factor in cases of hearing loss^29^. The results reported in this study may reflect the lack of awareness among the youth on the issues related to noise and its effects.

This study looked into only the habits of wearing earphones and frequenting places where loud music was played. Nonetheless, other studies have analyzed other habits (such as playing sports, musical instruments, working out at gyms etc) connected to young people^8^, ^30^. However, the habit of listening to music while wearing earphones is more common among young people. The findings in this study confirm this data and reveal that the number of adolescents wearing earphones is greater than the number of young people frequenting places where loud music is played, as also seen in the literature^31^, ^32^. This habit is growing in popularity among adolescents as is the possible risk it poses to hearing.

## CONCLUSION

According to the results described in this study, a significant portion of the tested subjects had altered transient evoked and distortion product otoacoustic emissions in at least one of their ears. The amplitudes recorded were higher among females and in right ears in both tests, and failed results were more prevalent among males. Most teens wear earphones and frequent places where loud music is played.
